# Usefulness of Imaging Response Assessment after Irreversible Electroporation of Localized Pancreatic Cancer—Results from a Prospective Cohort

**DOI:** 10.3390/cancers13122862

**Published:** 2021-06-08

**Authors:** Rasmus V. Flak, Rune V. Fisker, Niels H. Bruun, Mogens T. Stender, Ole Thorlacius-Ussing, Lars J. Petersen

**Affiliations:** 1 Department of Gastrointestinal Surgery, Aalborg University Hospital, DK-9000 Aalborg, Denmark; mogens.stender@rn.dk (M.T.S.); otu@rn.dk (O.T.-U.); 2 Department of Clinical Science, Aalborg University, DK-9220 Aalborg Øst, Denmark; ljp@dadlnet.dk; 3 Clinical Cancer Research Center, Aalborg University Hospital, DK-9000 Aalborg, Denmark; 4 Department of Nuclear Medicine and Radiology, Aalborg University Hospital, DK-9000 Aalborg, Denmark; rvf@rn.dk; 5 Unit of Clinical Biostatistics, Aalborg University Hospital, DK-9000 Aalborg, Denmark; nbru@rn.dk

**Keywords:** RECIST, progression, positron emission tomography, metabolic tumor volume, total lesion glycolysis, metastasis, systemic response, local response

## Abstract

**Simple Summary:**

Irreversible electroporation (IRE) is a novel therapy that is being studied for the treatment of nonmetastatic pancreatic cancer. The current methods for evaluating the treatment response after IRE have been adapted from the Response Evaluation Criteria in Solid Tumors (RECIST). However, it is uncertain whether these methods are appropriate, because the methods have not been validated. The aim of the current study was to evaluate the correlation between survival time and the most commonly used imaging assessment methods on FDG-PET/CT scans. We confirmed that the Response Evaluation Criteria in Solid Tumors (RECIST) are correlated with survival, when applied as intended. However, no correlation was found when the often-used lesion-level method was used. FDG-PET-derived data did not provide any benefit over conventional CT data. Several novel methods for lesion-level analysis were explored.

**Abstract:**

(1) Background: Irreversible electroporation (IRE) is a nonthermal ablation technique that is being studied in nonmetastatic pancreatic cancer (PC). Most published studies use imaging outcomes as an efficacy endpoint, but imaging interpretation can be difficult and has yet to be correlated with survival. The aim of this study was to examine the correlation of imaging endpoints with survival in a cohort of IRE-treated PC patients. (2) Methods: Several imaging endpoints were examined before and after IRE on ^18^F-fluorodeoxyglucose positron emission tomography (PET) with computed tomography. Separate analyses were performed at the patient and lesion levels. Mortality rate (MR) ratios for imaging endpoints after IRE were estimated. (3) Results: Forty-one patients were included. Patient-level analysis revealed that progressive disease (PD), as defined by RECIST 1.1, is correlated with a higher MR at all time intervals, but PD, as defined by EORTC PET response criteria, is only correlated with the MR in the longest interval. No correlation was found between PD, as defined by RECIST, and the MR in the lesion-level analysis. (4) Conclusions: Patient-level PD, as defined by RECIST, was correlated with poorer survival after IRE ablation, whereas no correlations were observed in the lesion-level analyses. Several promising lesion-level outcomes were identified.

## 1. Introduction

Pancreatic cancer (PC) is one of the deadliest and most challenging common cancers to treat. Its incidence is projected to increase significantly, especially in non-Western countries [1]. The only chance of long-term survival is radical surgical resection, which is not possible for most patients due to widespread metastasis at the time of diagnosis or involvement of major blood vessels in the surrounding area [2]. Recent oncological and surgical advances, including combination chemotherapy regimens, e.g., FOLFIRINOX, radiotherapy, and coeliac axis resection, have led to increased survival and resection rates, but surgical resection remains impossible in a substantial number of patients [3,4].

Irreversible electroporation (IRE) is a novel nonthermal ablation technique that has been applied in patients with nonmetastatic locally advanced unresectable PC (LAPC) and patients with isolated local recurrence to obtain local tumor control or even tumor shrinkage [5,6]. Despite the theoretical benefits of IRE, the efficacy of this treatment remains to be established, as there is only low-level evidence available to date [7]. However, initial unrandomized controlled prospective trial studies are promising and indicate that the treatment could be efficacious and may induce tumor shrinkage to a resectable stage in some cases [8,9,10,11].

When introducing new ablative therapies, it is often straightforward to provide evidence of safety and feasibility, whereas in the absence of randomized controlled trials, it can be difficult to answer the following questions: Is the treatment efficacious, and how do we evaluate the response to the treatment? Imaging outcomes have been widely adopted as a surrogate marker of treatment efficacy after IRE and other ablative treatments in locally advanced PC but can be difficult to interpret [12,13]. In a recent systematic review, it was demonstrated that there are theoretical problems in applying the commonly used imaging assessment strategies in local ablative treatments in PC patients (e.g., response evaluation criteria in solid tumors (RECIST) [14]) and that there is a lack of evidence correlating the imaging outcomes with survival after IRE [12].

Furthermore, many trials of IRE ablation have reported RECIST only at the lesion- level [12]. The omission of patient-level endpoints is not backed by evidence, may introduce misclassification of the tumor stage, and does not allow an assessment of whether the therapy inadvertently promotes metastasis.

When assessing a therapeutic response by RECIST (version 1.1), all lesions and lymph nodes are categorized as either measurable or non-measurable [14]. To be measurable, a lesion must be accurately measurable and at least 10 mm in size (on computed tomography (CT) imaging) in at least one dimension. At baseline, up to five target lesions (two per organ) are chosen for response evaluation, depending on how many organs are involved. The longest diameter (short axis for lymph nodes) of all target lesions is measured and summarized. When assessing the response after therapy, the patient will fall into one of four categories, depending on the changes in target and non-target lesions. Complete response (CR) is defined as a complete disappearance of target lesions and a regression of any lymph nodes (target or non-target) to below 10 mm in the short axis. Partial response (PR) is defined as a ≥30% decrease in the sum of diameters compared to the baseline. Progressive disease (PD) is characterized as a ≥20% increase in the sum of the diameters of target lesions and at least a 5 mm absolute increase compared to the lowest sum of diameters at any prior evaluation or the appearance of new lesions. Stable disease (SD) is defined as no changes allowing for categorization as PR or PD.

One of the major problems in applying this in a PC ablation context is that by using RECIST strictly, the ablated lesion falls into the category of unmeasurable disease, which only allows for categorization as CR, nonprogressive disease (non-PD), or PD in subsequent scans [14]. CR is not relevant in an ablation context because of the post-ablative scarring and cancer-associated fibrosis, which is apparent on post-ablative imaging. Therefore, it is not possible to characterize any positive changes (i.e., PR). Guidelines developed by the Society of Interventional Radiologists (SIR) try to remedy this by recommending using the first post-ablative scan as the baseline for response assessment. However, the correct categorization of the response is dependent on the assessment of the ablative efficacy, i.e., whether the ablation has completely eradicated all tumor cells at the ablation site [15]. Additionally, the assessment of ablative efficacy is difficult in pancreatic tumors, as the tumors have a high degree of fibrosis, which can be difficult to distinguish from actual tumor tissue. These interpretation difficulties are analogous to the difficulties in assessing resectability after aggressive chemotherapy regimens [16].

Given these issues, it seems likely that functional imaging technics, such as positron emission tomography (PET), could give valuable information about the actual tumor burden and not just cancer-associated fibrosis. Several distinct objective response assessment criteria are commonly used in assessing PET-derived data. These criteria use cut-off points of differences in uptake values of radiotracers to distinguish between CR, PR, SD, and PD and assess both target and remote lesions but are otherwise largely analogues to RECIST.

The aim of the current study was to examine the correlations between imaging outcomes and survival after IRE using established patient-level methods and lesion-level analysis. In addition, we sought to examine whether functional imaging is better at distinguishing between response categories than morphological imaging. To our knowledge, this is the first clinical trial examining the correlation between imaging outcomes and survival after IRE using both functional and morphological imaging techniques in PC patients.

## 2. Materials and Methods

The study was performed in a prospective cohort of PC patients treated with IRE at our institution. Survival and safety data from a subset of the same cohort of patients have been published previously [17].

### 2.1. Patient Recruitment and Selection

Patients were recruited from the treating institution or by referral from other Danish hospitals between October 2013 and March 2019. Patients were eligible to enter the study if they had localized (nonmetastatic) pancreatic ductal adenocarcinoma and were not candidates for surgery due to unresectable arterial/venous involvement according to the National Comprehensive Cancer Network (NCCN) guidelines [18]. Patients with resectable tumors and severe comorbidities contraindicating resection were also allowed to enter the trial.

All patients were preoperatively assessed by ^18^F-fluorodeoxyglucose (^18^F-FDG) PET with contrast-enhanced CT. Patients with unequivocal metastatic lesions at baseline were excluded prior to IRE treatment. Further exclusion criteria were age <18 years, life expectancy <3 months, persistent atrial fibrillation, implanted electronic devices or metal stents near the ablation zone, and severe allergies to anesthetic agents or other essential medications. All decisions to include a patient were determined by the local pancreatic multidisciplinary team (MDT) conference to ensure that resection was not possible. Patients were referred to another Danish HPB center for a second opinion if any doubt remained about resectability. Upfront chemotherapy, based on the oncologist’s choice, was encouraged in all patients unless they were not medically fit to receive it. Chemotherapy was paused at least 4 weeks prior to IRE.

### 2.2. Treatment Protocol

IRE treatments were performed in situ using an ultrasound-guided approach by an expert interventional radiologist. The ablations were carried out using the commercially available NanoKnife™ System (Angiodynamics, Queensbury, NY, USA). Pulse delivery was synchronized with the refractory period of the electrocardiogram to minimize the risk of cardiac arrythmias. Needle electrodes were placed percutaneously in the anterior–posterior direction. Needle placements were aimed at bracketing the tumor with a 5 to 10 mm margin of healthy tissue using two to six needles. The needles were placed in parallel with uniform depth and a maximum deviation of 5°. Twenty test pulses were given between each needle pair to establish tissue impedance and to ensure proper energy delivery. The aim was to reach 30 amperes between each needle pair. Afterward, 70 additional pulses were applied for each needle pair to achieve proper irreversible electroporation. One or more pullbacks and/or rearrangements were performed in patients with large tumors to electroporate the entire tumor.

### 2.3. Imaging Protocol

^18^F-FDG PET/CT was performed according to institutional procedures, which were in line with the European Association of Nuclear Medicine procedure guidelines for oncological PET exams [19]. The mean dose of ^18^F-FDG was 347 (range 172 to 400) MBq. Blood glucose levels were below 11 mmol/L in all patients. The PET/CT scan was acquired approximately 60 min after tracer injection. CT was performed as diagnostic CT with intravenous iodinated contrast (Iomeron^®^ 400 mg iodine/mL). The upper abdomen was scanned before contrast injection, in the pancreatic parenchymal phase and in the portal venous phase for optimal lesion detection, vessel involvement, and metastatic disease. Whole-body images were obtained using either a VCT Discovery True 64 PET/CT system (GE Healthcare, North Richland Hills, TX, USA) or a Siemens Biograph mCT Flow 64 PET/CT system (Siemens Healthineers, Erlangen, Germany). The patients were scanned from the base of the skull to the upper thigh, and PET images were acquired in 3D mode. The PET images were reconstructed using attenuation correction with an ordered subset expectation–maximization algorithm. For the Siemens Biograph mCT Flow, 64 PET/CT time-of-flight and point-spread functions were applied.

### 2.4. Imaging Analysis

All PET/CT images were independently reviewed by two readers (a radiologist and a nuclear medicine physician) with extensive experience with PET/CT and were reported at the lesion level (at the pancreatic bed). The number of remote pathological lesions was registered. Any discrepancy in interpretation among the readers was solved by consensus.

Automatically calculated, quantitative analyses of the ^18^F-FDG PET/CT images were performed by a hybrid imaging specialist (RVF) using dedicated software (SyngoVia VB40; Siemens Healthineers, Erlangen, Germany). Pathological lesions at the pancreatic bed were characterized by (1) the maximum standardized uptake value (SUVmax), (2) the metabolic tumor volume (MTV) with an SUV of >2.5 threshold, and (3) total lesion glycolysis (TLG). The MTV and TLG were analyzed using a threshold-based automatic volume of interest (VOI). TLG was representative of metabolic activity throughout the entire tumor and was calculated by multiplying the MTV and the mean SUV (SUVmean) of the MTV.

### 2.5. Imaging Endpoints

^18^F-FDG PET/CT scans were performed prior to treatment (baseline) and every 3 months after treatment. Imaging beyond 6 months was censored due to low numbers and due to confounding interventions after this timepoint, i.e., surgical resection or retreatment with IRE. Several endpoints were defined prior to image analysis.

Patient-level categorization as PD or non-PD was performed in accordance with RECIST 1.1 and the European Organization for Research and Treatment of Cancer (EORTC) PET response criteria [14,20]. PD according to RECIST was defined as a ≥20% increase in ablated tumor diameter and/or an increase in the number of CT-apparent suspected metastases. PD according to EORTC PET response criteria was defined as a ≥25% increase in SUVmax at the ablated lesion or an increase in the number of PET-positive suspected metastases.

Lesion-level categorization as PD was performed based on RECIST (self-termed “local RECIST”), defined as a ≥20% increase in the tumor diameter of the ablated lesion. Moreover, categorization of PD was established for functional imaging parameters (i.e., SUVmax, MTV, and TLG) and was defined as a ≥25% increase in the values based on the EORTC criteria cut-off.

Because of the low number of patients, additional exploratory analyses were performed at the lesion level to examine whether any correlations exist between the unestablished lesion-level outcome measures and patient survival.

### 2.6. Statistical Analysis

Poisson regression with the Huber/White sandwich estimator was used to estimate mortality rate ratios (MRRs) [21]. Ninety-five percent confidence intervals were provided for all estimates. The baseline imaging date was considered the entry date, and the date of death was considered the exit date. Patients alive on 21 March 2020 were censored from this date. Two analyses were performed. In the first analysis, the correlations between patient- and lesion-level categorical outcomes and survival were analyzed. In the second analysis, potential lesion-level imaging parameters were treated as continuous variables to examine whether there is a correlation between these measures and survival. This was chosen because categorization of continuous variables comes at the cost of statistical power. Multivariate analysis was omitted due to the small sample size. All data were analyzed using Stata version 16 (StataCorp LLC, College Station, TX, USA). To examine whether the SIR guidelines are more suitable for long-term follow-up, the time intervals were divided into two groups based on the comparison scan used (baseline or first postablative scan) for illustrative purposes.

## 3. Results

**Figure 1:**
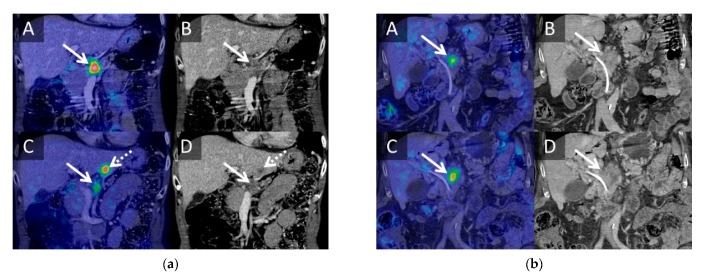
Representative ^18^FDG PET/CT images before and after IRE. (**a**) Sixty-one-year-old female with a T4 tumor in the head/body of the pancreas without nodal metastases. The patient was treated with three different chemotherapy regimens (FOLFIRINOX, XELOX, and gemcitabine) prior to inclusion (eight series in total). The patient was referred to IRE due to progression on chemotherapy. PET/CT images were performed before IRE (**A**,**B**) and 3 months after IRE (**C**,**D**). The patient had regression of SUVmax (9 -> 3.5 SUV_bw_), MTV (9 -> 1.86 cm^3^), TLG (37.51 -> 5.41 SUV_bw_cm^3^), and tumor size (23 -> 20 mm) at the primary tumor (solid arrow) but developed a PET-avid metastasis in liver segment III (dotted arrow). The patient received seven additional series of chemotherapy (XELOX and gemcitabine) after the 3-month scan and survived for 21 months after IRE ablation. (**b**) Seventy-seven-year-old male with a T4 tumor in the head/body of the pancreas with local nodal metastases. The patient was treated with six series of GEMCAP chemotherapy prior to inclusion. The patient was referred to IRE treatment due to progression on chemotherapy. PET/CT images were performed prior to IRE (**A**,**B**) and 3 months after IRE (**C**,**D**). The patient had increased SUVmax (5.27 -> 6.04 SUV_bw_), MTV (5.09 -> 10.66 cm^3^), TLG (16.31 -> 34.73 SUV_bw_ cm^3^), and tumor size (25 -> 33 mm) of the primary tumor (arrow) after IRE ablation. The patient did not receive any chemotherapy after IRE ablation, because of intolerance. The patient survived for 10 months after IRE ablation.

Forty-one patients entered the trial after selection. Most included patients (*n* = 33) had locally advanced PC (LAPC). Baseline demographics are provided in Table 1. All patients were treated using the ultrasound-guided percutaneous approach except for one patient who was treated using an open approach as the tumor could not be visualized ultrasonographically on the day of treatment. All patients were scanned at baseline, while 35 and 22 patients were scanned at 3 and 6 months, respectively. An illustrative example of PET/CT images before and after IRE is available in Figure 1. All raw imaging data are plotted in Supplementary File S1.

### 3.1. Patient-Level Analysis

PD according to RECIST 1.1 was significantly correlated with an increased mortality rate (MR) during all time intervals (Table 2). The estimated MRR was highest in the time interval where the scan for comparison was the first post-ablative scan (MRR = 7.075). In comparison, PD according to the EORTC PET response criteria was not significantly correlated with the MR in the early interval (baseline to 3 months), and the estimated correlation during the baseline-to-6-month interval was lower than the RECIST-based estimation (MRR = 1.829 vs. 2.546). The MRR could not be estimated for EORTC-based PD in the 3- to 6-month interval, as all patients were categorized as having progressive disease.

### 3.2. Lesion-Level Analysis

PD determined by local RECIST was not significantly correlated with the MR in any of the examined time intervals (Table 3). Similarly, none of the functional parameters tested as categorical outcomes were significantly correlated with the MR, but the MTV and TLG had higher estimated MRRs than local RECIST and trended toward statistical significance, especially in the time interval using the first post-ablative scan for comparison.

### 3.3. Exploratory Lesion-Level Analysis

Lesion-level analysis of tumor size increase, as a continuous variable, was found to be significantly correlated with the MR in the baseline-to-6-month interval (MRR = 2.992) and in the interval where the first post-ablative scans were used for comparison (MRR = 4.024). In the analysis of functional lesion-level imaging outcomes, it was found that the SUVmax increase was not significantly correlated with the MR in any of the intervals (Table 4). However, both MTV and TLG increases were significantly correlated with an increased MR in the baseline-to-6-month interval and were slightly better correlated during the 3- to 6-month interval. The two outcomes were not correlated in the early (baseline to 3 months) interval. Cross comparison between the four continuous variables (tumor size, SUVmax, MTV, and TLG) was not possible because the estimated MRR is dependent on the unit of the individual imaging outcome measure.

## 4. Discussion

In this study, by examining the correlation between imaging outcomes and survival in patients undergoing IRE for localized pancreatic cancer, we found that PD at the patient level, as defined by RECIST 1.1, is correlated with worse survival. Likewise, PD according to EORTC was correlated with a higher MR in the baseline-to-6-month interval but not in the early time interval and, furthermore, was unable to differentiate patients at all in the 3- to 6-month interval. The estimated MRRs for RECIST 1.1 were higher than those assessed by the EORTC, and thus, patient-level assessment by ^18^F-FDG PET did not provide any benefit over morphological imaging in distinguishing response categories.

Although widely adopted in studies of local ablations in LAPC, local RECIST was not significantly correlated with the mortality rate [12]. However, this may be due to the small sample size and the cut-off point (20% increase) because a significant correlation was found when tumor size measures were input as continuous variables in Poisson regressions.

In the absence of evidence for using local RECIST, several functional imaging parameters were tested. Increases in the MTV and TLG were found to be correlated with the MR, but both parameters failed to correlate with the MR in the early post-operative period (baseline to 3 months), which suggests that other inter-related factors may have confounded the functional imaging parameters—most likely post-ablative inflammation. PD, as defined by a ≥25% increase in the MTV or TLG, was not significantly correlated with the MR, but it is reasonable to assume that this may be due to the sample size because both parameters trended toward significance and both were correlated in the exploratory analysis as continuous variables.

Based on these findings, it seems likely that functional imaging could have value in lesion-level analysis but is susceptible to confounding and needs validation before adoption as a marker of efficacy in clinical trials.

In general, a trend of better differentiation between PD and non-PD, i.e., a higher MRR, was noted in the analysis using the first postablative scan as the comparison scan. This was seen in most assessments at the lesion level and when using RECIST 1.1 at the patient level and thus provides evidence supporting the practice as proposed by the SIR [15].

Although by no means definitive, these findings provide a preliminary evidence base for objective response assessment in future trials of IRE in LAPC. However, there are several separate issues in this area of research that should be addressed. First, how do we evaluate the ablative efficacy, i.e., whether the ablation has covered the entirety of the tumor and whether complete eradication of tumor cells is to be expected? Second, how do we evaluate any positive outcomes, i.e., response, at the lesion level if complete eradication of tumor cells has not taken place?

Answering these questions is beyond the scope of this study, as nearly all patients in this cohort experienced local progression and thus evidently did not experience complete eradication of tumor cells at the ablation site. This is most likely because IRE is simply not able to eradicate all tumor cells in the ablated area; thus, the therapy is merely cytoreductive. Other authors have tested imaging techniques that might be more suitable to distinguish between complete or incomplete ablation, but no evidence of clinical benefit has been provided to validate these techniques [13].

Based on the difficulties in distinguishing tumor tissue from fibrosis, particular care should also be taken when assessing resectability after IRE ablation, as several accounts of successful resections have been reported after IRE [17,22,23,24,25]. It is possible that functional imaging techniques could provide valuable information in this regard but should be validated further. Arguably, if there is any doubt about the post-ablative resectability on imaging, a surgical exploration should be performed.

The timing of post-ablative scans should also be considered carefully, as early follow-up scans could potentially be beneficial as comparison scans. However, it was not possible to examine this in the current cohort of patients, as this was not part of the study design. Furthermore, there is some evidence that suggests that the IRE effect may be more long-lasting, extending for weeks or even months [26,27]. Therefore, scans in the early post-ablative period could potentially be confounded by ongoing effects.

To our knowledge, this is the first study correlating PET/CT parameters with clinical benefit after IRE ablation. However, one study has previously correlated CT imaging outcomes after IRE with survival in localized PC [28]. The researchers found that local recurrence within 6 months after IRE was highly correlated with a worse survival outcome (HR 7.71) compared to patients with no apparent local recurrence during this period. These findings are consistent with the results presented in this study.

The primary limitation of this study was the small number of participants, as this prevented the development of a multivariate model that could have accounted for major confounders such as performance status at baseline, adjuvant chemotherapy given during follow-up, age, and baseline tumor size. It is possible that results of the study were influenced by the immediate interpretation of the scans, because early indications of progression may have led to early post-ablative chemotherapeutic treatment, which, in turn, may have affected the outcomes. Furthermore, due to changes in PET scanners during the trial, it was not possible to apply the more modern PET Response Criteria in Solid Tumors (PERCIST 1.0), as the older scanner lacked EARL accreditation.

In contrast to some of the published studies on IRE, patients in this study were not required to have stable disease prior to IRE treatment [29,30]. Most patients in the cohort were already experiencing local disease progression prior to the IRE procedure, and this, while making the study more externally valid, probably skewed the results toward progression in the short term when compared to other studies.

## 5. Conclusions

Patient-level PD based on RECIST 1.1. correlated with poorer survival after IRE in localized PC after IRE ablation. ^18^F-FDG PET results were likely confounded by post-ablative inflammation in the early post-ablative period and did not provide any benefit over morphological imaging in patient-level analysis. Lesion-level analysis is inappropriate at this point in time, but several promising parameters should be examined in future trials.

## Figures and Tables

**Table 1:** Study demographics.

Age	Years	Mean (Range)	67.6	(50.3; 82.8)
Sex	Female	*n* (%)	19	(46.3%)
	Male		22	(53.7%)
Indication	LAPC	*n* (%)	33	(80.5%)
	ILR		4	(9.8%)
	MIPC		4	(9.8%)
Pre-IRE chemotherapy	Yes	*n* (%)	35	(85.4%)
	No		6	(14.6%)
Post-IRE chemotherapy	Yes	n (%)	25	(61.0%)
	No		16	(39.0%)

LAPC, locally advanced pancreatic cancer; ILR, isolated local recurrence after previous resection; MIPC, medically inoperable pancreatic cancer; IRE, irreversible electroporation.

**Table 2:** Univariate regression analysis of patient-level outcomes.

Outcome	Comparison Scan	Time Interval	MRR	Lower 95% CI	Upper 95% CI
Progressive disease (RECIST 1.1)	Baseline	0–3 months	**2.646**	1.253	5.587
		0–6 months	**2.546**	1.033	6.273
	Post-ablative	3–6 months	**7.075**	2.944	17.000
Progressive metabolic disease (EORTC)	Baseline	0–3 months	1.420	0.537	3.757
		0–6 months	**1.829**	1.108	3.020
	Post-ablative	3–6 months	*	*	*

Statistically significant results in bold. * Results not shown because all patients were categorized as progressors; MRR, mortality rate ratio; CI, confidence interval; CT, computed tomography; PET, positron emission tomography; RECIST 1.1, Response Evaluation Criteria in Solid Tumors version 1.1; EORTC, European Organization for Research and Treatment of Cancer PET response criteria.

**Table 3:** Univariate regression analysis of categorical lesion-level outcomes.

Outcome	Comparison Scan	Time Interval	MRR	Lower 95% CI	Upper 95% CI
Local RECIST (20% increase)	Baseline	0–3 months	1.073	0.494	2.332
		0–6 months	1.852	0.711	4.821
	Post-ablative	3–6 months	1.373	0.454	4.155
SUVmax (25% increase)	Baseline	0–3 months	0.665	0.297	1.489
		0–6 months	1.149	0.440	3.004
	Post-ablative	3–6 months	2.050	0.837	5.021
MTV (25% increase)	Baseline	0–3 months	1.972	0.863	4.505
		0–6 months	2.658	0.890	7.941
	Post-ablative	3–6 months	2.678	0.977	7.339
TLG (25% increase)	Baseline	0–3 months	2.209	0.992	4.920
		0–6 months	2.658	0.890	7.941
	Post-ablative	3–6 months	2.333	0.841	6.467

**Table 4:** Univariate regression analysis of exploratory continuous variables at the lesion level.

Outcome	Comparison Scan	Time Interval	MRR	Lower 95% CI	Upper 95% CI
Tumor diameter on CT (per cm increase)	Baseline	0–3 months	2.151	0.977	4.735
		0–6 months	**2.992**	1.238	7.234
	Post-ablative	3–6 months	**4.024**	1.580	10.246
SUVmax (per SUV_bw_ increase)	Baseline	0–3 months	1.001	0.996	1.005
		0–6 months	1.027	0.887	1.190
	Post-ablative	3–6 months	2.050	0.837	5.021
MTV (per cm^3^ increase)	Baseline	0–3 months	0.996	0.960	1.034
		0–6 months	**1.015**	1.007	1.024
	Post-ablative	3–6 months	**1.018**	1.010	1.025
TLG (per SUV_bw_cm^3^ increase)	Baseline	0–3 months	1.000	0.993	1.008
		0–6 months	**1.004**	1.002	1.007
	Post-ablative	3–6 months	**1.005**	1.003	1.007

Statistically significant results in bold.

## Data Availability

Data available on request due to restrictions, e.g., privacy or ethical.

## Supplementary Materials

The following are available online at https://www.mdpi.com/article/10.3390/cancers13122862/s1, Figure S1: Plots of individual patient imaging data.
