# Supplementary figures and images for: Usefulness of Imaging Response Assessment after Irreversible Electroporation of Localized Pancreatic Cancer—Results from a Prospective Cohort
Source: Cancers (Basel). 2021 Jun 8;13(12):2862. doi: 10.3390/cancers13122862 (PMC8226515; doi:10.3390/cancers13122862)

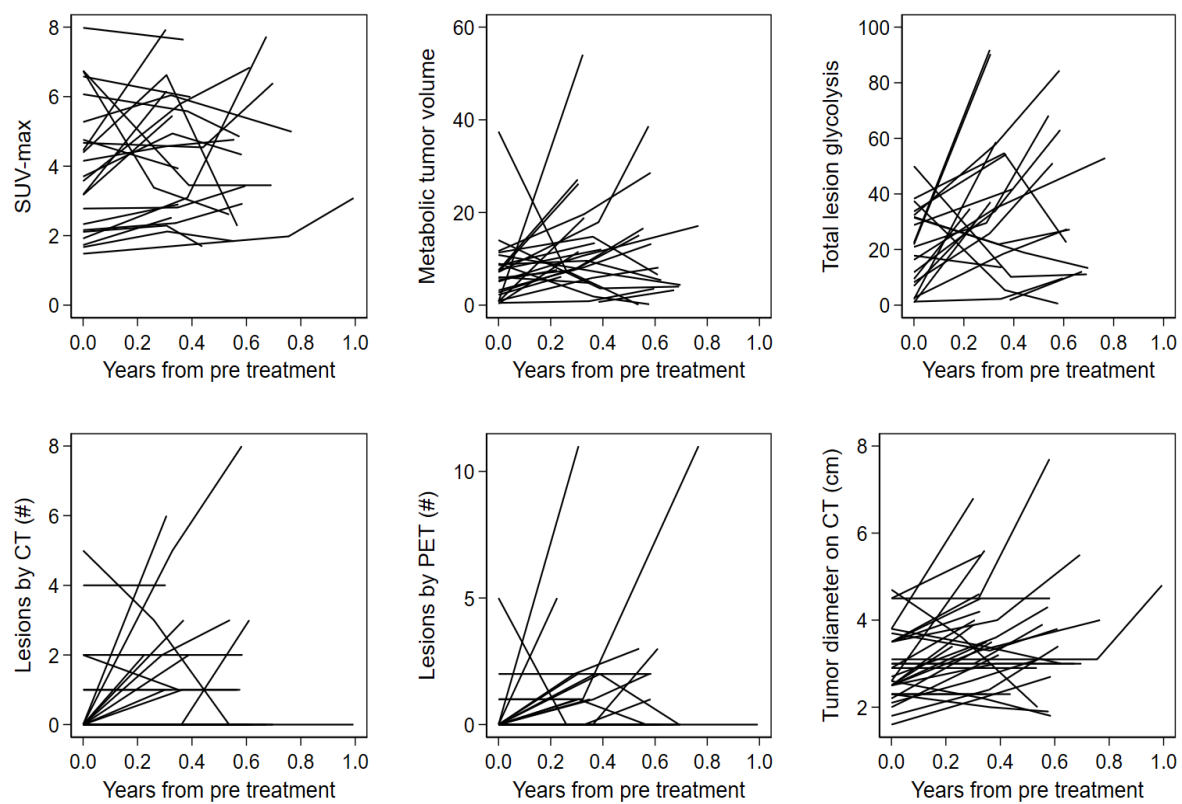

**Figure S1.** Plots of individual patient imaging data.

Supplement: Supplementary file 1 [file cancers-13-02862-s001.zip › cancers-1205889-supplementary.pdf]
